# Targeting JAK/STAT signalling with cold atmospheric plasma: a potential therapy for systemic lupus erythematosus

**DOI:** 10.1017/erm.2026.10042

**Published:** 2026-03-24

**Authors:** Xiaofeng Dai, Yuting Fan, Zizheng Huang

**Affiliations:** 1 National Local Joint Engineering Research Center for Precision Surgery & Regenerative Medicine, Shaanxi Provincial Center for Regenerative Medicine and Surgical Engineering, First Affiliated Hospital of Xi’an Jiaotong University, Xi’an, China; 2 Department of Gastroenterology, The Affiliated Hospital of Guizhou Medical University, Guiyang, China

**Keywords:** cold atmospheric plasma, Janus kinase–signal transducer and activator of transcription, Systemic lupus erythematosus, therapeutics

## Abstract

Systemic lupus erythematosus (SLE) is a complex autoimmune disease with heterogeneous multi-organ manifestations and poorly understood pathogenesis. The variable therapeutic outcomes and potential for cross-indication treatments underscore the need to identify pivotal disease-driving signalling pathways for developing innovative and safer regimens. By categorizing the pathogenesis of SLE into three interconnected stages involving over-activated immune response, skewed cytokine homeostasis and impaired debris clearance, and by analysing public clinical data, this review posits that the Janus kinase–signal transducer and activator of transcription axis is a central driver of SLE pathogenesis. Accordingly, it explores the potential of cold atmospheric plasma to modulate this pathway for therapeutic benefit that requires further experimental and clinical validations.

## Introduction

Systemic lupus erythematosus (SLE) is a chronic autoimmune disease afflicting approximately 3.4 million individuals worldwide (Ref. 1). It predominantly affects women of the childbearing age, with a female-to-male ratio of approximately 9:1 (Ref. 2). The clinical manifestations of SLE are highly heterogeneous, ranging from mild skin lesions to the failure of multiple organs, and are characterized by the over-representation of anti-nuclear antibody (ANA), anti-double-stranded DNA (anti-dsDNA) antibody, anti-Smith (anti-Sm) antibody, antiphospholipid (aPL) antibody and anti-β2-glycoprotein (aβ2GPI) antibody (Ref. 1). The onset and activity of SLE can be assessed using metrics such as the American College of Rheumatology (ACR) classification criteria issued in 2019 SLE (Ref. 3), the SLE Disease Activity Index (SLEDAI) and its updated versions (Refs 4, 5). The diagnostic accuracy may be complicated by comorbidities accompanied with SLE, including, e.g., cancers, cardiovascular diseases, bone disorders and neuropsychiatric diseases (Ref. 1).

SLE remains a clinically challenging autoimmune disorder in which chronic inflammation, immune dysregulation and variable therapeutic responses collectively limit durable disease control. Recent advances across inflammatory medicine underscore that pathologic immune activation is mechanosensitive and metabolically plastic, offering opportunities for intervention beyond conventional immunosuppression: mechanotransduction pathways such as Piezo1 can regulate disease-relevant cellular phenotypes under physical cues (e.g., ultrasound), (Ref. 6) while sirtuin-mediated stress responses such as SIRT6 act as endogenous brakes on endotoxin-driven endothelial inflammation (Ref. 7). In parallel, translational efforts continue to refine inflammation-linked clinical stratification tools and outcomes research, exemplified by biomarker-based sepsis screening models (Ref. 8) and real-world evaluations of anti-infective regimens in high-risk resistant infections, (Ref. 9) emphasizing the importance of precision approaches when inflammation and infection risks converge. Emerging therapeutic concepts further highlight the value of energy-assisted and biofilm-disruptive modalities, (Ref. 10) metabolism-targeted anti-inflammatory strategies that reverse inflammatory senescence (Ref. 11) and extracorporeal immune-modulating platforms such as lymphoplasmapheresis in refractory neuroinflammatory disease (Ref. 12).

The pathogenesis of SLE is subjected to the genetic/epigenetic predisposition, and primed by both environmental triggers and hormonal factors (Refs 13–16). Though over-activated B cells and immune responses have been considered playing indispensable roles in priming SLE, the pathogenesis of SLE involves a complicated crosstalk among diversified pathways such as signals relayed through type I interferon (IFN), (Ref. 17) Toll-like receptor (TLR), (Ref. 18) the nucleotide-binding oligomerization domain (NOD), (Ref. 19) the phosphoinositide 3 kinase (PI3K)/a serine/threonine protein kinase (AKT) axis, (Ref. 20) mitogen-activated protein kinase (MAPK)/extracellular signal regulated kinase (ERK) pathway, (Ref. 21) mammalian target of rapamycin (mTOR) pathway (Ref. 22) and the Janus kinase (JAK)/signal transducer and activator of transcription (STAT) cascade (Refs 23–26). These have complicated our understanding on SLE pathogenesis, rendering current therapeutics for SLE management largely rely on canonical medications including, e.g., antimalarial agents such as hydroxychloroquine, (Ref. 27) immunomodulators such as glucocorticoid, (Ref. 28) methotrexate, (Ref. 29) azathioprine, (Ref. 30) mycophenolate, (Ref. 31) voclosporin, (Ref. 32) biologics such as belimumab, (Ref. 33) anifrolumab, (Ref. 34) telitacicept (Ref. 35) and B cell depletion therapies such as rituximab (Ref. 31) and CD19-targeting chimeric antigen receptor (CAR) T cells (Ref. 5). Conventional SLE regimens, including antimalarials, corticosteroids, non-steroidal immunosuppressants, as well as biologics and B cell depletion, often incur significant toxicities like retinopathy, infections and organ toxicity, and elicit heterogeneous patient responses (Refs 36–39). Besides, the clinical use of CAR-T therapies is largely hindered by the limited cell source, complicated production process and high cost (Ref. 40). Importantly, certain therapeutics effective in treating SLE may paradoxically trigger drug-induced lupus in a subset of susceptible patients, a clinically significant albeit infrequent phenomenon. For example, infliximab, initially designed to ameliorate tumour necrosis factor (TNF)-related reactions for treating inflammatory bowel disease (Ref. 41) and rheumatic arthritis, was effective in managing some SLE patients (Ref. 42) yet triggered lupus-like symptoms among some rheumatic arthritis carriers (Refs 43, 44). Therefore, it is of paramount clinical relevance and urgency to gain an in-depth understanding of SLE pathogenesis towards the establishment of advanced strategies for accurate SLE management.

Motivated by complicated manifestation and pathogenesis of SLE as well as its urgent unmet therapeutic needs, this study identified JAK/STAT as the central pathway orchestrating the signalling landscape potentiating SLE, and proposed the promising benefits of cold atmospheric plasma (CAP) for SLE patients that takes actions via, at least partially, targeting this molecular axis. In this review, we posit that JAK/STAT is a central pathogenic axis in SLE and explore the potential of CAP to modulate this pathway.

## SLE pathogenesis

The pathogenic mechanisms of SLE remain to be fully unveiled. As antibodies such as ANA, anti-Sm, anti-dsDNA, aPL and aβ2GPI are exclusively produced by plasma cells (terminally differentiated B cells) of SLE patients, over-activated auto-reactive B cells and immune response have been considered with critical roles in potentiating SLE (Ref. 36).

Besides, CD4^+^ T helper (Th) cells participating in the differentiation and proliferation of B cells, such as Th1, Th2, Th17 and T regulatory (Treg) cells, are also involved in SLE initiation and progression. These CD4^+^ T cells secrete different cytokines to orchestrate the immune homeostasis, the distribution of which, once skewed, may trigger SLE and accelerate its activity. While Treg cells are responsible for controlling immune tolerance, which can suppress over-activated Th1/2/17 cells, Th17 cells activation is known to be involved in autoimmune diseases. Th1 and Th2 cells form a pair that suppresses each other, with the activation of Th1 and Th2 cells being associated with cell immunity and humoral immunity, respectively. Therefore, in the case of SLE, skewed cytokine distribution favouring Th2 and/or Th17 is a sign of SLE pathogenesis, and that in favour of Th1 and Treg plays an opposite role (Ref. 45). Recently, several novel sub-cohorts of Th cells have been characterized including, e.g., T follicular helper (Tfh), T peripheral helper (Tph) and follicular regulatory T (Tfr) cells (Refs 46–50). According to our current knowledge, these cells largely function in the Th2 linkage. While Tfh and Tph serve as activators of B cells for enhanced SLE activity, Tfr cells suppress the activity of Tfh cells for attenuated SLE syndrome (Refs 49, 51).

Attributing to these over-activated immune cells and abnormal cytokine landscape, excessive auto-antibodies are produced by B cells that bind to autoimmune antigens such as autologous nucleic acids, phospholipids and histones to form immune complexes. These complexes, once deposited on organs or tissues, activate inflammatory reactions, leading to deteriorated protein structure and organ functionality. Damaged cells or tissues result in improved generation of auto-antigens, forming a vicious cycle that drives SLE progression.

Various signalling pathways have been implicated in altered immune reactions among SLE patients. For instance, IFN-mediated signalling primes both innate and adaptive immune responses, over-activation of which is involved in SLE development (Ref. 17). The TLR axis relays immune activation signals via sensing nucleic acids; (Ref. 18) B cell receptor (BCR) and T cell receptor (TCR) pathways participate in SLE pathogenesis via directly regulating the differentiation, activation and survival of primary immune cells implicated in SLE progression (Refs 36, 52) and different members of the JAK and STAT families take actions in different stages during SLE progression (Refs 23, 45).

Besides immunopathology, oxidative stress is another well-documented feature of SLE, as patients typically exhibit elevated ROS production (Ref. 53). Specifically, the accumulation of DNA-damaging markers like 8-hydroxydeoxyguanosine has been associated with the onset (Ref. 54) and activity (Ref. 55) of SLE, where overt ROS drives SLE pathogenesis by promoting inflammatory and cellular damage (Ref. 56).

## JAK/STAT is central for SLE pathogenesis

### JAK/STAT orchestrates diverse pathways for promoted SLE pathogenesis

**Figure 1.**
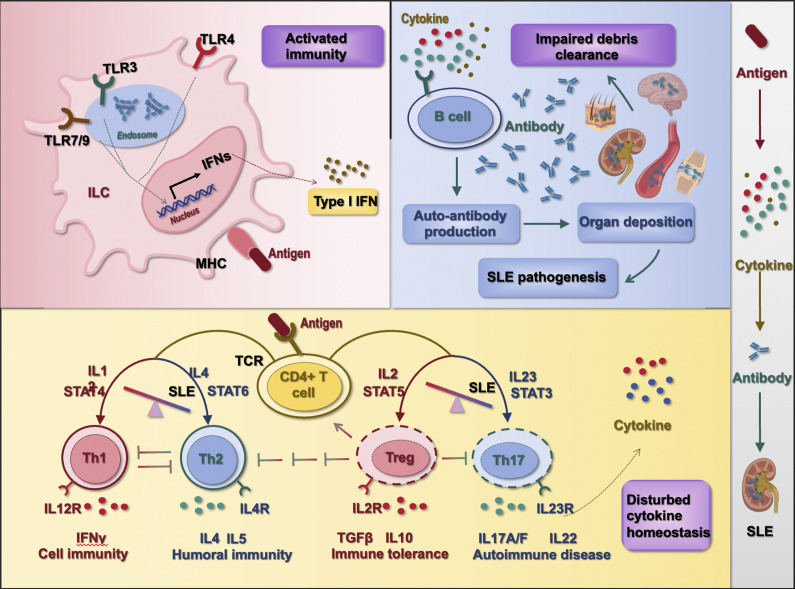
Critical stages orchestrating the pathogenesis of SLE. The pathogenesis of SLE can be classified into three critical stages, i.e., ① over-activated immunity, ② disturbed cytokine homeostasis and ③ impaired debris clearance. Over-activated immunity leads to over-represented antigens that result in dis-regulated polarization of CD4^+^ T cells into Th1, Th2, Th17 and Treg cells and consequently disturbed cytokine homeostasis. Th1 cells are characteristic of secreting IFNγ and being activated by interleukin-12 (IL12) and STAT4; Th2 cells are featured by producing IL4 and IL5 among others and are inductive by IL4 and STAT6; Th17 cells are known for producing IL17A/F and IL22 that can be stimulated by IL23 and STAT3; Treg cells predominantly produce transforming growth factor beta (TGFβ) and IL10, and can be primed by IL2 and STAT5. Imbalanced cytokine homeostasis can further lead to overt production of auto-antibodies that, once deposited in organs without timely clearance (as a result of impaired debris clearance machinery), prime SLE pathogenesis.

For clarity, we propose that the myriad pathways in SLE can be grouped into three key pathogenic stages (Figure 1): an initial immune hyperactivation (stage I), a resulting cytokine imbalance (stage II) and failure of debris clearance leading to autoantigen persistence (stage III) (Ref. 45). Genetic predispositions (like complement deficiencies and HLA associations) and environmental factors (e.g., UV exposure and infections) set the stage for these immunologic disturbances, though a full discussion is beyond the review’s scope. Many disparate pathways can be grouped into these three functional stages of lupus immunopathology. For instance, through aggregate and single variant association testing, 21 pathways were characterized using 958 SLE patients and 1026 healthy individuals, most of which can be matched into this three-stage framework (Ref. 57).

Among these signalling axes, retinoic acid-inducible gene (RIG)-I-like signalling pathway (hsa04622), TLR signalling pathway (hsa04620), NOD-like receptor signalling pathway (hsa04621) and C-type lectin receptor signalling pathway (hsa04625), representing four major functionally distinct clades of pattern recognition receptors (PRRs) responsible for pathogen-associated molecular pattern (PAMP) detection, (Ref. 58) contribute to SLE pathogenesis by driving the ‘initial immune hyperactivation’ in the innate immune system (Stage I).

Th1 and Th2 cell differentiation (hsa04658) and Th17 cell differentiation (hsa04659) are primary CD4^+^ subsets identified, leading to ‘cytokine imbalance’ in SLE patients. Complement and coagulation cascades (hsa04610) represent the primary mechanism associated with ‘failure of debris clearance’ among lupus carriers. These pathways, which function within the adaptive immune system, correspond to Stages II and III of the pathogenesis model, respectively.

Other signalling axes identified from this study (Ref. 57) can also be matched into this categorization framework. Specifically, viral protein interaction with cytokine and receptor (hsa04061), Fc epsilon RI signalling (hsa04664), cytosolic DNA-sensing pathway (hsa04623), antigen processing and presentation (hsa04612), TCR signalling (hsa04660), NK cell-mediated cytotoxicity (hsa04650), intestinal immune network for immunoglobulin A (IgA) production (hsa04672), haematopoietic cell lineage (hsa04640) and the osteoclast differentiation pathway (hsa04380) can be classified into ‘stage I’ given their direct roles in activating the innate or adaptive immune systems (note that osteoclast belongs to the monocyte/macrophage family); and nuclear factor κB (NF-κB) signalling (hsa04064) can be grouped into this category given the responsiveness of NF-κB to diverse stimuli including PRRs, TNF receptors, TCRs and BCRs (Ref. 59). Cytokine–cytokine receptor interaction (hsa04060) and TNF pathway (hsa04668) belong to ‘stage II’ as reflected by the names of these signalling axes and the membership of TNF in the family of cytokines.

**Figure 2.**
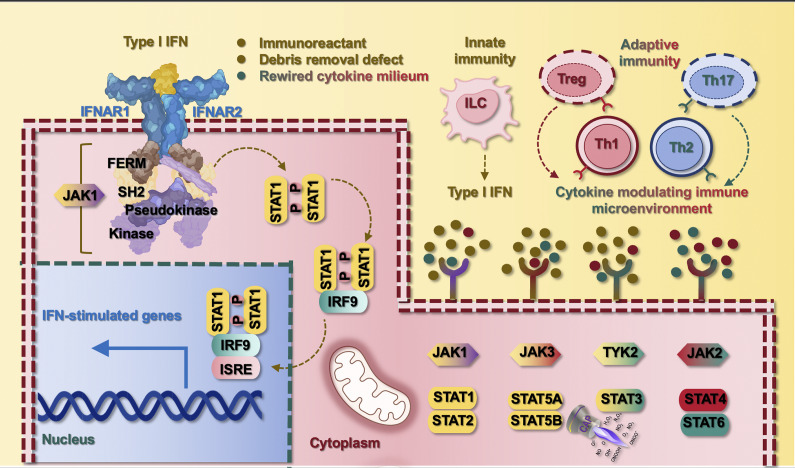
JAK/STAT orchestrates SLE pathogenesis and the antagonist role of CAP against this axis. SLE pathogenesis can be initiated or promoted by three main sources, i.e., immune stimulants, defective debris removal machinery and rewired cytokine milieu. While immune stimulants and inefficient debris removal may activate the innate immune system for enhanced production of type I IFNs from innate lymphocyte cells, rewired cytokine milieu reflects the skewed subsets of CD4^+^ T cells (i.e., Th1, Th2, Th17, Treg) that are associated with abnormal cytokine profiles and altered JAK/STAT signalling. Type I IFNs can also bind the extracellular domain of its receptor to initiate the signalling cascade mediated by JAK and STAT family members. JAK is composed of four family members, i.e., JAK1, JAK2, JAK3 and TYK2. JAK1, JAK3 and TYK2 primarily respond to type I IFNs and Th1, Th2 and Th17 cytokines; and JAK2 largely responds to Th1 and Th2 cytokines. STAT includes seven family members, i.e., STAT1, STAT2, STAT3, STAT4, STAT5A, STAT5B and STAT6. STAT1, STAT2 and STAT5, which are mainly responsible for relaying type I IFNs signalling, STAT4 and STAT6 respond primarily to Th1 and Th2 cytokines, respectively, which form a pair controlling the Th1/Th2 switch and the homeostasis between cellular and humoral immunity. STAT3 can form homodimers or heterodimers with STAT1 in response to Th2 cytokines and participate in debris deposition besides its other roles. Take JAK1 as an example, it contains four domains, i.e., four-point-one protein, ezrin, radixin, moesin (FEBR), Src homology 2 (SH2), pseudokinase and kinase; in response to type I IFN stimulation, JAK1 phosphorylates STAT1 that forms dimers and is translocated into the cell nucleus to initiate the transcription of IFN-stimulated genes. CAP is known to target STAT3-mediated signalling, suggesting its feasibility in treating SLE.

The sole signalling axis, difficult to uniquely classify into the identified three-class framework, is the JAK/STAT pathway (hsa04630) that participates in almost all stages of the signalling network during SLE pathogenesis. As one of a handful of pleiotropic cascades capable of transducing a multitude of signals towards cytokine homeostasis in mammals, (Ref. 60) various JAK and STAT family members form diversified combinations to participate in all stages during SLE pathogenesis. For example, while STAT1, STAT2 and STAT5 mainly participate in type I IFNs signalling in ‘stage I’, STAT4 and STAT6 respond primarily to Th1 and Th2 cytokines in ‘stage II’, and STAT3 forms dimers with STAT1 to contribute to debris deposition in ‘stage III’; and JAK1, JAK3 and tyrosine kinase 2 (TYK2) have a comparatively larger responsive spectrum than JAK2 to fine-tune the responsive magnitude of stimuli from each source (Figure 2).

### JAK/STAT family members participate in different SLE pathogenesis stages

The JAK/STAT signalling pathway, conserved among metazoans, has been implicated in the pathogenesis of autoimmune diseases, including SLE, and proposed as a marker of SLE severity (Ref. 61).

JAKs contain a carboxy-terminal catalytic or kinase domain, a pseudokinase domain, a Src homology 2 (SH2) domain and an amino-terminal band four-point-one ezrin radixin moesin (FERM) domain, where the kinase and pseudokinase domains take the catalytic activities, and FERM and SH2 domains associate JAKs with the intracellular Box1 and Box2 domains of cytokine receptors (Ref. 62). During JAK/STAT signalling, cytokine receptor ligation leads to JAK auto-phosphorylation followed by the phosphorylation of tyrosine residues in the intracellular domain of the receptor that recruits cytoplasmic STATs through the SH2 domain and converts latent STATs to the active state. (Ref. 63) Phosphorylated STATs form homo or hetero-dimerizers and are translocated to the nucleus to regulate the transcription of the target genes.

The JAK family has four members, i.e., JAK1, JAK2, JAK3 and TYK2. JAK1 and JAK3 transduce signals from γ-chain cytokines and type I interferons, whereas JAK2 pairs with receptors for cytokines like GM-CSF and IL-3/5, and TYK2 is required for IL-12/23 and IFNα/β signalling. Specifically, JAK1 is sensitive to the stimuli of type I IFNs, as well as cytokines polarizing Th1 (i.e., IL2, IL7, IL15), Th2 (i.e., IL4, IL9, IL10) and Th17 (i.e., IL11, IL15, IL7, IL21) cells, among others; (Refs 52, 64–67) JAK3 shares a similar cytokine response profile with JAK1 (i.e., IL2, IL4, IL7, IL9, IL15, IL21); JAK2 participates in signalling involving cytokines produced by Th1 (i.e., granulocyte macrophage colony-stimulating factor, abbreviated as GM-CSF) and Th2 cells (i.e., IL3, IL5), out of others; and TYK2 is a downstream player relaying type I IFN-mediated signals and responds to cytokines driving the differentiation of Th1 cells (i.e., IL12), Th2 cells (i.e., IL6, IL10, IL13) and Th17 cells (i.e., IL23) (Refs 68–71) (Figure 1).

Six members of the STAT family have been so far identified, i.e., STAT1 to STAT6. In most cases, a specific STAT is activated by a given cytokine, yet all STATs can be stimulated by the major cytokines that have been under extensive investigation, and some cytokines can activate almost all STATs under certain conditions (Ref. 72). Specifically, STAT1 plays a significant role in mediating the anti-proliferative effects of type I/II IFNs, (Ref. 73) the protein level of which is enriched in circulating CD4^+^ T cells of SLE patients (Refs 61, 74). The essential role of STAT1 in relaying type I/II IFNs signals suggested its importance in bridging the innate and adaptive immune responses and the central role in driving SLE pathogenesis. Similar to STAT1, STAT2 is stimulated by type I IFNs that form heterodimers with STAT1 to take action (Refs 75, 76). STAT4, predominantly expressed in lymphoid tissues, can be activated by Th1-preferable pro-inflammatory cytokines such as IL12 and type I IFNs. STAT6, on the other hand, can be stimulated by Th2-favourable anti-inflammatory cytokines such as IL4 (Ref. 77). The responsive profiles of STAT4 and STAT6 make them a pair controlling the Th1/Th2 switch and the balance between cellular and humoral immunities (Ref. 78). Besides, STAT5, sub-classifiable into STAT5A and STAT5B, can be activated by prolactin (Refs 79–81) and has been proposed to mark SLE severity given the positive correlation between the accumulated STAT5 phosphorylation levels in CD4^+^ T cells and time-adjusted cumulative SLE activity (Ref. 82). STAT3 can form homodimers or heterodimers with STAT1 in response to IL6, which is largely produced by Th2 cells (Refs 83, 84) and mediates localized deposition of the complement C3, (Ref. 85) where the renal level of C3 is negatively associated with SLE severity (Ref. 86). Taken together, STAT1, STAT2 and STAT5 are mainly responsible for relaying type I IFNs among other functionalities; STAT4 and STAT6 are largely involved in modulating the cytokine microenvironment; and STAT3 participates in debris deposition besides its other roles (Figure 2).

## SLE therapeutics targeting JAK/STAT signalling

As outlined in the preceding section, the JAK/STAT pathway serves as a central nexus for numerous SLE-relevant immune signals. Accordingly, strategies such as inhibitors of JAK or STAT family members have been established for managing SLE (Ref. 87). Several JAK inhibitors have been used or are being examined in the clinics for SLE treatment. For instance, upadacitinib, an oral JAK1 inhibitor, has been reported to be capable of reducing flares with well-accepted tolerance among SLE patients through 48 weeks of use in a phase II clinical trial (NCT03978520) (Ref. 88). Filgotinib, another JAK1 inhibitor, has demonstrated its therapeutic benefits by two phase II clinical trials in treating lupus membranous nephropathy (NCT03285711) (Ref. 89) and moderate-to-severe cutaneous lupus erythematosus, abbreviated as CLE (NCT03134222) (Ref. 90). GSK2586184, another JAK1 inhibitor, showed efficacy in treating SLE patients (Ref. 91). Baricitinib, being an oral selective inhibitor of JAK1/2, has been investigated by several phase II/III clinical trials for SLE treatment (NCT03616912, NCT03616964 and NCT02708095), with a rapid SRI-4 response being documented at Week 24 (Refs 92–94). However, clinical trials of baricitinib for SLE were terminated for futility in some clinical trials (NCT03843125 and NCT03616912), and the drug consequently did not receive FDA approval for this indication. Ruxolitinib, another JAK1/2 inhibitor, improved the clinical symptoms of SLE patients carrying haemophagocytic lymphohistocytosis (Ref. 95). Tofacitinib, a specific inhibitor of JAK1/JAK2/JAK3, exhibited desirable efficacy without obvious adverse effects in treating SLE patients from a phase I clinical trial (NCT02535689) (Ref. 96). Deucravacitinib, an inhibitor of TYK2, lowered the disease severity of SLE patients without demonstrated safety concerns, including deaths, opportunistic infections and major adverse cardiovascular or thrombotic events (NCT03252587) (Ref. 97). By inhibiting JAK1/2/3 and TYK2, gusacitinib and delgocitinib have been used in two clinical trials targeting SLE (phase I, NCT06238531) (Ref. 98) and subacute CLE (phase II, NCT03958955), (Ref. 99) respectively (Table 1).

**Table 1.** Therapeutics targeting JAK/STAT signalling for SLE treatment approved or under clinical investigation

Commercial name	Medication type	Trial number	Phase	Status	Primary outcome	References
Upadacitinib	JAK1 inhibitor	NCT05843643	III	Ongoing	NA	(Ref. 153)
		NCT03978520	II	Completed	Upadacitinib reduced flares with well-accepted tolerance among SLE patients through 48 weeks of use.	(Ref. 88)
Filgotinib	JAK1 inhibitor	NCT03134222	II	Completed	In patients with moderate-to-severe CLE, 68.8% achieved a ≥ 5-point improvement in CLASI-A score at Week 12 and 50% achieved a ≥ 50% improvement.	(Ref. 90)
		NCT03285711	II	Completed	Filgotinib demonstrated therapeutic benefits in treating lupus membranous nephropathy	(Ref. 89)
GSK2586184	JAK1 inhibitor	NCT01777256	II	Terminated	At interim analysis, GSK2586184 showed no significant effect on mean interferon transcriptional biomarker expression. The study was declared futile and recruitment was halted at 50 patients.	(Ref. 91)
Baricitinib	JAK1/2 inhibitor	NCT03616912	III	Terminated	Primary endpoint in this study was met for the 4 mg baricitinib group. However, key secondary endpoints were not met. This study terminated due to insufficient evidence to support a positive benefit.	(Ref. 93)
		NCT03616964	III	Completed	There was no difference in the primary efficacy outcome of the proportion of SRI–4 responders at Week 52 between participants who received baricitinib 4 mg and placebo. None of the major secondary endpoints, including glucocorticoid tapering and time to first severe flare, were met.	(Ref. 94)
		NCT02708095	II	Completed	Baricitinib resulted in a rapid and sustained significant decrease in anti-dsDNA antibodies and IgG levels in the 4 mg group at Weeks 12 and 24.	(Ref. 92)
		NCT03843125	III	Terminated	This study was terminated due to insufficient evidence to support a positive benefit.	(Ref. 154)
		NCT05686746	II/III	NA	NA	(Ref. 155)
		NCT05432531	III	Ongoing	NA	(Ref. 156)
Ruxolitinib	JAK1/2 inhibitor	NCT04908280	II	Completed	Ruxolitinib cream applied for 12 weeks lowered IGA scores, relieved itch and pain and reduced erythema in discoid lupus patients.	(Ref. 157)
		NCT06261021	II	Ongoing	NA	(Ref. 158)
		Clinical case study	NA	NA	Ruxolitinib improved the clinical symptoms of SLE patients carrying haemophagocytic lymphohistocytosis	(Ref. 95)
Tofacitinib	JAK1/2/3 inhibitor	NCT02535689	I	Completed	Tofacitinib demonstrated clinical efficacy and was well-tolerated in patients with SLE.	(Ref. 96)
		NCT05048238	I	Completed	Tofacitinib decreased CLASI and SLEDAI–2 K score.	(Ref. 159)
		NCT03288324	I/II	Completed	Eight weeks of tofacitinib (5 mg bid) led to a decrease in the CLASI activity score among young SLE patients with moderate-to-severe skin disease.	(Ref. 160)
Deucravacitinib	TYK2 inhibitor	NCT03920267	II	Completed	NA	(Ref. 161)
		NCT04857034	II	Ongoing	NA	(Ref. 162)
		NCT05620407	III	Ongoing	NA	(Ref. 163)
		NCT05617677	III	Ongoing	NA	(Ref. 164)
		NCT03252587	II	Completed	Deucravacitinib lowered the disease severity of SLE patients without demonstrated safety concerns, including deaths, opportunistic infections and major adverse cardiovascular or thrombotic events.	(Ref. 97)
Gusacitinib	JAK1/2/3, TYK2 inhibitor	NCT06238531	I	Withdraw	Drug company discontinuing study.	(Ref. 98)
Delgocitinib	JAK1/2/3, TYK2 inhibitor	NCT03958955	II	Terminated	This study was terminated due to recruitment challenges.	(Ref. 165)
		Clinical case study	NA	NA	Topical delgocitinib application for 2 weeks led to complete resolution of rashes in the subacute cutaneous lupus erythematosus patient, with no flare-ups over 6 months.	(Ref. 99)
Fludarabine	STAT1 inhibitor	NCT00001676	I/II	Completed	A short course of low-dose fludarabine and cyclophosphamide induced long-lasting disease remissions among LN patients, but with bone marrow toxicity.	(Ref. 101)
Artesunate	STAT3 inhibitor	NCT03214731	IV	NA	NA	(Ref. 166)

CLASI: Cutaneous Lupus Erythematosus Disease Area and Severity Index; IGA: Investigator’s global assessment; JAK: Janus kinase; LN: lupus nephritis; SLEDAI: Systemic Lupus Erythematosus Disease Activity Index; SRI: SLE Responder Index; STAT: signal transducer and activator of transcription; TYK: tyrosine-protein kinase 2.

Inhibitors of STATs have also been used or investigated for SLE treatment. A short course use of low-dose fludarabine (an inhibitor of STAT1 (Ref. 100)) and cyclophosphamide induced long-lasting disease remissions among lupus nephritis (LN) patients, but bone marrow toxicity from a phase I/II study (Ref. 101). Static, a small molecule inhibiting STAT3, delayed the onset of LN *in vivo* (Ref. 102). By down-regulating the phosphorylation levels of JAK2 and STAT3, artesunate (a semisynthetic derivative from artemisinin) demonstrated its therapeutic efficacy in treating lupus-prone MRL/lpr mice (Ref. 103) (Table 1). Clinical examples of inhibitors of other STAT family members in the treatment of SLE are not yet available but deserve extensive investigations. These include, e.g., SH-4-54 (a small molecule targeting the SH2 domains of both STAT3 and STAT5) with demonstrated efficacy in treating multiple types of cancers, (Refs 104, 105) STAT5-IN-1 (a specific inhibitor of STAT5) with proposed feasibility in treating atherosclerosis (Ref. 106) and AS1517499 (a STAT6 inhibitor) with *in vivo* evidence of alleviating the syndrome of asthma (Ref. 107).

Despite promising clinical progress, the immunosuppressive nature of JAK/STAT inhibitors raises concerns about infection risk and long-term safety in SLE, a population already vulnerable to infections. Ensuring long-term safety thus remains a challenge, highlighting the imperative to develop innovative therapeutic modalities that can sustainably modulate this pathway.

## CAP as a promising SLE therapeutics

To identify novel therapeutic avenues, this section explores the potential of CAP as an innovative therapeutic modality for SLE. CAP belongs to the fourth state of matter, is composed of a cocktail of free radicals such as hydroxyl radical (OH•), superoxide anion radical (O_2_•), singlet oxygen (_1_O^2^), hydrogen peroxide (H_2_O_2_), ozone (O_3_), nitric oxide (NO) and peroxynitrite (OONO^−^), (Refs 108, 109) and is typically operated at the room temperature (37 ~ 44 °C) (Ref. 110). CAP can be prepared by imposing a high electric field on the air or the process gas, typically helium (He), argon (Ar) or their mixtures with oxygen (O_2_). This resulted in the establishment of various reliable CAP generation sources (based on three primary discharge modes, i.e., dielectric barrier discharge, spark discharge and corona discharge) that can deliver mild yet effective doses of reactive oxygen and nitrogen species (RONS).

CAP has demonstrated diverse therapeutic effects in a plethora of clinical issues, such as wound healing, (Ref. 111) haemostasis (Ref. 112) and sterilization (Ref. 113). Ever since 2007, when the first report on the role of CAP in selectively killing cancer cells was published, the specificity of CAP in ablating transformed cells without impairing the functionality of normal cells has been demonstrated in a large spectrum of malignant cells (Refs 109, 114–117). Intense pre-clinical investigations on the molecular mechanisms followed, which demonstrated the distinctive traits of CAP as a promising onco-therapy such as its multimodality, (Ref. 118) cell selective properties, (Refs 119–123) immune-modulating abilities (Refs 124, 125) and synergistic effects with conventional therapeutic agents, especially immune checkpoint blockade therapy (Refs 126, 127). These established medical applications of CAP, especially its cell-selective and immune-modulating properties, raise the question of whether CAP could be repurposed for autoimmune conditions such as SLE.

With improved understanding of the biomedical features and working mechanisms of CAP, the ‘redox homeostasis theory’ has been proposed to explain the selectivity of CAP against transformed cells, which associates the redox modulatory roles of CAP with the distinct redox homeostatic levels possessed by cells of different physiological and pathological states (Ref. 110). Imbalanced cellular redox levels underpin pathological inflammation that not only occurs in cancer cells but also in autoimmune diseases. This has extended the medical efficacy of CAP to other complex disorders, including several autoimmune diseases that, in most cases, are currently of no effective yet safe cure. For instance, the innovative use of CAP for treating vitiligo, (Ref. 128) psoriasis (Refs 129–131) and Alzheimer’s diseases (Refs 132, 133) has been documented or implicated. Thus, though not having been reported, the possible benefits of CAP in treating SLE may be forecasted and are worthy of being tested in the wet lab.

It is important to emphasize that the roles of CAP in modulating the redox homeostasis and the JAK/STAT axis are dose-dependent (Ref. 134). This is because the chemical species within CAP and the outcomes it may achieve for a given biological application are controlled by parameters such as the amount of energy applied, the type and pressure of the processing gas, the discharge mode and treatment duration, as well as cell features such as the availability and types of surface receptors (Refs 110, 135). Such a dose-dependent nature is of paramount importance as it makes CAP possible to sensitize cancer cells to immune therapies on one hand and attenuate the immune responses in autoimmune disorders on the other hand, which builds up the basis for us to discuss the possible unparalleled therapeutic benefits of CAP for treating SLE.

At the molecular level, CAP may take actions and benefit SLE patients via modulating JAK/STAT signalling. Given that all three stages of SLE pathogenesis converge on the JAK/STAT axis, investigating the role of CAP in this pathway could provide insight into its potential efficacy in countering overactivated immune responses (stage I), regulating cytokine distribution (stage II) and promoting clearance of apoptotic debris (stage III), each of which plays a critical role in SLE development. It has been reported that CAP suppressed JAK/STAT3 signalling in healthy human epithelial MCF10A cells and African green monkey kidney Vero E6 cells, (Ref. 136) whereas it activated the JAK/STAT1 axis in macrophages towards the active state within the tumour microenvironment of melanoma (Ref. 137). In addition, immunomodulatory agents targeting JAK/STAT signalling such as methotrexate (138) and fludarabine (Ref. 101) have been demonstrated to be feasible for arresting cancers such as childhood leukaemia and breast cancers (Refs 29, 139, 140) and have been proposed to be beneficial for treating autoimmune diseases such as rheumatoid arthritis and SLE (Refs 101, 141, 142). This evidence further supports the concept of treating cancers and autoimmune diseases via attenuating JAK/STAT signalling, as well as the possible benefits of CAP to SLE patients (Figure 2).

In addition to evidence demonstrating CAP’s impact on the JAK/STAT axis, a convergent pathway in all key stages of SLE pathogenesis, direct evidence also indicates that CAP modulates immune cells and inflammatory processes in a dose- and context-dependent manner. For instance, CAP has been shown to induce monocyte differentiation into M1 macrophages, accompanied by increased secretion of TNF-α and IL-6 (Ref. 143). Consistent with this, CAP restored imbalanced macrophage polarization by expanding the M1 population in triple-negative breast cancer, (Ref. 144) stimulated macrophages in metastatic solid tumours to hinder cancer progression (Ref. 145) and promoted M1 macrophage differentiation via the JAK2/STAT1 axis (Ref. 137). Beyond macrophages, CAP activated bone marrow-derived dendritic cells (DCs) of the innate immune system, (Ref. 146) inhibited Th17 cell differentiation *in vitro* (Ref. 146) and enhanced adaptive immune responses in a melanoma animal model (Ref. 147). Moreover, CAP significantly accelerated skin wound healing by modulating the immune microenvironment and attenuating inflammation (Ref. 148). In rheumatoid arthritis, CAP effectively reduced the production of inflammatory mediators such as NF-κB and IL-6, as well as destructive factors like RANKL and MMP-3, thereby slowing disease progression by suppressing tumour-like features and inflammation in fibroblast-like synoviocytes (Refs 149, 150).

Despite the therapeutic promise of CAP for SLE, a significant technical hurdle exists that may limit its direct clinical application. SLE is a systemic disease often involving internal organs like the kidney and heart, whereas CAP is inherently a localized therapy, currently used mainly for skin conditions and surface sterilization. To overcome this limitation, indirect strategies utilizing CAP-generated reactive species stored in other states, like liquid, gel or solid, are required. For instance, plasma-activated medium, where the medium can be any liquid such as water, phosphate-buffered saline or Ringer’s solution, has been commonly used for CAP-associated medical investigations. Thus, CAP may potentially be delivered via plasma-activated infusions to affect systemic immunity, perhaps via treating blood in an extracorporeal circuit, analogous to photopheresis or via intravenous administration to modulate immune cells.

This necessitates a thorough evaluation of the safety profile of CAP in chronic use to prevent unintended tissue damage or exacerbated oxidative stress. This concern is underscored by its dose-dependent effects on redox balance, as demonstrated in a study where 2-minute argon CAP exposure induced a pro-oxidant state in rat blood plasma, whereas a 1-minute exposure stimulated the plasma’s antioxidant capacity (Ref. 151). This paradoxical effect stems from the dual role of free radicals: at physiological levels, they are crucial signalling molecules regulating mitochondrial function and immune responses; in excess, they overwhelm antioxidant defences, causing oxidative damage to cellular components (Ref. 152). Consequently, the misuse of CAP risks causing tissue injury or immunosuppression, making the identification of a precise therapeutic window a critical research priority. Future validation requires experiments such as treating SLE patient-derived peripheral blood mononuclear cells with CAP to monitor inflammatory cytokine release and STAT pathway activation. Concurrently, developing technologies for rapid, clinical assessment of cellular redox status from blood samples is essential to enable the safe translation of CAP therapy for SLE.

## Conclusion

We emphasize that perturbed redox homeostasis is the fundamental cause of the immune signalling abnormalities that potentiate SLE, a process centrally orchestrated by the JAK/STAT signalling pathway. We forecast the promising use of CAP, alone or as an adjuvant approach, for treating SLE if used appropriately regarding the dosage and treatment duration, which may function via modulating the JAK/STAT axis.

We propose that the possible use of CAP in the treatment of SLE does not mean that it surpasses existing SLE therapeutics, but rather considers that it substantiates the current treatment portfolio and extends the use of CAP to the field of SLE. This may pave the way for novel therapeutic approaches in addressing unresolved clinical challenges of SLE by, e.g., synergizing conventional strategies with CAP, leveraging the power of the most abundant form of matter in the universe (the plasma state) and establishing innovative therapeutics (not limited to CAP) for treating SLE via restoring cell redox homeostasis. This reflects our overarching thesis: that therapeutic advancement lies in fully leveraging natural dynamics and adhering to inherent biological laws. Going forward, conducting thorough preclinical assessments, in lupus immune cells, animal models and clinical trials, constitutes a critical next step for establishing the safety and efficacy profile of CAP therapy.
